# The Laws of War Relating to the Wounded

**Published:** 1898-05-28

**Authors:** 


### THE LAWS OF WAR RELATING TO THE WOUNDED.

In general it may be stated that the rights of war in respect to the enemy are to be measured by the object of the war. Until that object is attained the belligerent has, strictly speaking, a right to use every means necessary to accomplish the end for which he has taken up arms. The practice of the ancient world, and even the opinion of some modern writers on public law, made no distinction as to the means to be employed for this purpose. Even Bynkershoek and Wolff, who lived at the beginning of the eighteenth century, assert the broad principle that everything done against an enemy is lawful; that he may be destroyed, though unarmed and defenceless; that fraud, and even poison, may be employed against him; and that an unlimited right is acquired by the victor to his person and property. Such, however, was not the sentiment and practice of enlightened Europe at the period when they wrote, since Grotius had long before inculcated milder and more humane principles, which Vattel subsequently enforced and illustrated, and which are adopted by the unanimous concurrence of all the public jurists of the present age.

Formerly when nation attacked nation, the whole inhabitants of the hostile State were regarded as liable to the rigours of warfare; but at the present day, not only old people, women and children, are regarded as exempt from the violence of warfare, but also in general all those subjects of the hostile State who do not bear arms or who take no active part in hostilities; and also even those who follow the enemy's camp but take no part in hostilities—such as medical men, commissaries, and all who can be strictly classed as non-combatants. In the case of enemies rendered harmless by wounds or disease, the growth of humane feeling has long passed the simple requirement that they shall not be killed or ill-used, and has cast upon belligerents the duty of tending them so far as is consistent with the primary duty to their own wounded. But the care which the wounded of a defeated army thus obtain is necessarily inadequate to their wants, and the usefulness of surgeons on both sides is hampered by their liability to be detained as prisoners. A step of which the value in mitigating the unnecessary horrors of war cannot be over-estimated would therefore be made if a general and sufficiently full understanding were arrived at as to the treatment of sick and wounded, and of persons and things engaged in their service, which should give free scope, as far as the exigencies of war permit, to the action of everyone whom duty or charity may enlist in the mitigation of suffering. Under the Convention of Geneva of 1864 the great part of the European States bound themselves to observe a code framed with this object, and the accession of nearly all the civilised States of the world has converted its provisions into rules of overwhelming authority. This Convention was signed on behalf of Switzerland, Baden, Belgium, Denmark, Spain, France, Hesse-Darmstadt, Italy, Netherlands, Portugal, Russia, and Würtemburg, August 22nd, 1864, and is now in full force. The following Powers have also acceded to it: Argentine Republic, 1879; Austria, 1866; Bavaria, 1866; Bolivia, 1879; Bulgaria, 1884; Chili, 1879; Great Britain, 1865; Greece, 1865; Japan, 1887; Mecklenburg-Schwerin, 1865; Montenegro, 1875; Persia, 1874; Peru, 1880; the Pope, 1868; Roumania, 1874; Russia, 1867; Salvador, 1874; Saxony, 1866; Servia, 1876; Sweden and Norway, 1864; Turkey, 1865; United States, 1882.

The Articles are as follows:—

"Art. I. Ambulances and military hospitals shall be acknowledged to be neuter, and as such shall be protected and respected by belligerents so long as any sick or wounded may be therein. Such neutrality shall cease if the ambulances or hospitals should be held by a military force.

"Art. II. Persons employed in hospitals and ambulances, comprising the staff for superintendence, medical service, administration, transport of wounded, as well as chaplains, shall participate in the benefit of neutrality whilst so employed, and so long as there remain any wounded to bring in or to succour.

"Art. III. The persons designated in the preceding article may, even after occupation by the enemy, continue to fulfil their duties in the hospital or ambulance which they serve, or may withdraw in order to rejoin the corps to which they belong. Under such circumstances, when these persons shall cease from their functions, they shall be delivered by the occupying army to the outposts of the enemy.

"Art. IV. As the equipment of military hospitals remains subject to the laws of war, persons attached to such hospitals cannot, in withdrawing, carry away any articles but such as are their private property. Under the same circumstances an ambulance shall, on the contrary, retain its equipment.

"Art. V. Inhabitants of the country who may bring help to the wounded shall be respected, and shall remain free. The generals of the belligerent Powers shall make it their care to inform the inhabitants of the appeal addressed to their humanity, and of the neutrality which will be the consequence of it. Any wounded man entertained and taken care of in a house shall be considered as a protection thereto. Any inhabitant who shall have entertained wounded men in his house shall be exempted from the quartering of troops, as well as from a part of the contributions of war which may be imposed.

"Art. VI. Wounded or sick soldiers shall be entertained and taken care of, to whatever nation they may belong. Commanders-in-chief shall have the power to deliver immediately to the outposts of the enemy, soldiers who have been wounded in an engagement, when circumstances permit this to be done, and with the consent of both parties. Those who are recognised, after their wounds are healed, as incapable of serving, shall be sent back to their country. The others may also be sent back, on condition of not again bearing arms during the continuance of the war. Evacuations, together with the persons under whose directions they take place, shall be protected by an absolute neutrality.

"Art. VII. A distinctive and uniform flag shall be adopted for hospitals, ambulances, and evacuations. It must, on every occasion, be accompanied by the national flag. An arm badge shall also be allowed for individuals neutralised, but the delivery thereof shall be left to military authority. The flag and the arm badge shall bear a red cross on a white ground. (Turkey uses a red crescent)."

The following additional articles were signed at Geneva on October 20th, 1868, on behalf of Great Britain, Austria, Baden, Bavaria, Belgium, Denmark, France, Italy, Netherlands, North Germany, Sweden and Norway, Switzerland, Turkey, and Würtemberg. On July 22nd, 1870, it was stated by the Swiss Government that all those States on whose behalf the original convention had been signed had adhered to the additional articles, Rome and Spain excepted, but that Russia, whilst agreeing to the additional articles, proposed a supplement to Art. XIV., with the view of preventing the abuse to the distinguishing flag of neutrality. The chief articles are as follows:—

"Art. I. The persons designated in Article II. of the convention shall, after the occupation by the enemy, continue to fulfil their duties, according to their wants, to the sick and wounded in the ambulance or the hospital which they serve. When they request to withdraw, the commander of the occupying troops shall fix the time of departure, which he shall only be allowed to delay for a short time in case of military necessity.

"Art. III. Under the conditions provided for in Articles I. and IV. of the convention, the name (ambulance) applies to field hospitals and other temporary establishments, which follow troops on the field of battle to receive the sick and wounded.

"Art. V. In addition to Article VI. of the convention, it is stipulated that, with the reservation of officers whose detention might be important to the fate of arms and within the limits fixed by the second paragraph of that article, the wounded fallen into the hands of the enemy shall be sent back to their country, after they are cured, or sooner if possible, on condition, nevertheless, of not again bearing arms during the continuance of the war.

"Art. VI. The boats which, at their own risk and peril, during and after engagement, pick up the shipwrecked or wounded, or which, having picked them up, convey them on board a neutral or hospital ship, shall enjoy, until the accomplishment of their mission, the character of neutrality as far as the circumstances of the engagement and the position of the ships engaged will permit. The appreciation of these circumstances is entrusted to the humanity of all the combatants. The wrecked and wounded thus picked up and saved must not serve again during the continuance of the war.

"Art. VII. The religious, medical, and hospital staff of any captured vessel are declared neutral, and on leaving the ship may remove the articles and surgical instruments which are their private property.

"Art. VIII. The staff designated in the preceding article must continue to fulfil their functions in the captured ships, assisting in the removal of wounded made by the victorious party; they will then be at liberty to return to their country in conformity with the second paragraph of the first additional article.

"Art. IX. The military hospital ships remain under martial law in all that concerns their stores; they become the property of the captor, but the latter must not divert them from their special appropriation during the continuance of the war. The vessels not equipped for fighting which during peace the Government shall have officially declared to be intended to serve as floating hospital ships, shall, however, enjoy during the war complete neutrality, both as regards stores, and also as regards their staff, provided their equipment is exclusively appropriated to the special service on which they are employed.

"Art. XI. Wounded or sick sailors and sailors when embarked, to whatever nation they may belong, shall be protected and taken care of by their captors. Their return to their own country is subjected to the provisions of Article VI. of the Convention, and of the additional Article V.

"Art. XII. The distinctive flag to be used with the national flag, in order to indicate any vessel or boat which may claim the benefits of neutrality in virtue of the principles of this Convention, is a white flag with a red cross. Military hospital ships shall be distinguished by being painted white outside, with green strake.

"Art. XIII. The hospital ships which are equipped at the expense of the aid societies, recognised by the Government signing this convention, and which are furnished with a commission emanating from the Sovereign, who shall have given express authority for their being fitted out, and with a certificate from the proper naval authority that they have been placed under his control during their fitting out and on their final departure, and that they were then appropriated solely to the purpose of their mission, shall be considered neutral, as well as the whole of their staff. They shall be recognised and protected by the belligerents. They shall make themselves known by hoisting, together with their national flag, the white flag with a red cross. The distinctive mark of their staff, while performing their duties, shall be an armlet of the same colours. The outer painting of these hospital ships shall be white with red strake. These ships shall bear aid and assistance to the wounded and wrecked belligerents without distinction of nationality. They must take care not to interfere in any way with the movements of the combatants. During and after the battle they must do their duty at their own risk and peril. The belligerents shall have the right of controlling and visiting them; they will be at liberty to refuse their assistance, to order them to depart, and to detain them if the exigencies of the case require such a step. The wounded and wrecked picked up by these ships cannot be reclaimed by either of the combatants, and they will be required not to serve during the continuance of the war."

Conventions such as these for the care of the wounded, and the prohibition of explosive bullets in warfare, have done much, and they will do still more, to mitigate the frightful horrors of war.

